# Mechanotransduction Impairment in Primary Fibroblast Model of Krabbe Disease

**DOI:** 10.3390/biomedicines11030927

**Published:** 2023-03-16

**Authors:** Roberta Mezzena, Ambra Del Grosso, Roberto Maria Pellegrino, Husam B. R. Alabed, Carla Emiliani, Ilaria Tonazzini, Marco Cecchini

**Affiliations:** 1 NEST, Istituto Nanoscienze—CNR and Scuola Normale Superiore, Piazza San Silvestro 12, 56127 Pisa, Italy; 2 Department of Chemistry, Biology, and Biotechnologies, University of Perugia, 06123 Perugia, Italy

**Keywords:** mechanotransduction, Krabbe disease, focal adhesions, migration, autophagy

## Abstract

Krabbe disease (KD) is a genetic disorder caused by the absence of the galactosylceramidase (GALC) functional enzyme. No cure is currently available. Here, we investigate the mechanotransduction process in primary fibroblasts collected from the twitcher mouse, a natural KD murine model. Thanks to mechanotransduction, cells can sense their environment and convert external mechanical stimuli into biochemical signals that result in intracellular changes. In GALC-deficient fibroblasts, we show that focal adhesions (FAs), the protein clusters necessary to adhere and migrate, are increased, and that single-cell migration and wound healing are impaired. We also investigate the involvement of the autophagic process in this framework. We show a dysregulation in the FA turnover: here, the treatment with the autophagy activator rapamycin boosts cell migration and improves the clearance of FAs in GALC-deficient fibroblasts. We propose mechanosensing impairment as a novel potential pathological mechanism in twitcher fibroblasts, and more in general in Krabbe disease.

## 1. Introduction

Krabbe disease (KD) is a rare genetic lysosomal storage disorder caused by the mutation of the galactosylceramidase (GALC) gene. The GALC enzyme is widely expressed in the mammalian body and its absence determines a severe disease, involving myelin loss, neurodegeneration, and death in a few years. The main pathological features are linked to the accumulation of the cytotoxic sphingolipid psychosine (PSY), based on the so-called “psychosine hypothesis” [1]. PSY acts as a detergent and alters the biological membrane architecture [2,3]. Various effects are attributed to its accumulation in the nervous system: from demyelination and gliosis to membrane shedding enhancement [4] and endocytosis inhibition in neural cells. Enzyme replacement in vivo appears to be insufficient to reconstitute the homeostasis and remove PSY [5]. This could be presumably attributed to the involvement of several molecular pathways and organelles in PSY accumulation [6], such as PLA2 [7], mitochondria, Akt signalling [8], proapoptotic factor [9], inflammation and immune response [10,11], calcium signalling [12], and much more. Moreover, several findings are emerging in the literature concerning new aspects of KD’s pathogenesis that cannot be explained by PSY accumulation alone. This theory is evolving, and one of the latest aspects pointed out is that PSY is secreted in extracellular vesicles in the brain of the twitcher mouse (the natural KD murine model); however, substrate reduction therapy can increase lifespan but not rescue the phenotype [13]. Alterations were found in the structure and function of the brain endothelium of KD patients and twitcher mice, in vivo and in vitro, revealing an increased permeability of the vessels [14]; importantly, studies on zebrafish embryos [15] and on human endothelial cells [16] suggested that these effects were not PSY-dependent. Teixeira and colleagues [17] showed that in the twitcher mouse, there are fewer axons in the central and peripheral nervous system before the onset of demyelination, with impairment in the retrograde component of axonal transport and microtubule stability. Moreover, in KD patients-derived fibroblasts [18], lactosylceramide (another GALC substrate) metabolism is impaired, even in the absence of PSY accumulation. Thus, a clear picture of KD-related intracellular signalling is still far from being achieved.

We recently demonstrated for the first time that [19] autophagy is dysregulated in the twitcher mouse (GALC^−/−^), the spontaneous KD murine model. We found dysregulation of the autophagy markers p62 and LC3-II in the brain, sciatic nerve, and primary fibroblasts of GALC^−/−^ mice. This effect is not completely driven by PSY, because from the literature we know that GALC^−/−^ fibroblasts, either murine [19] or human [20], do not present significant PSY accumulation. Rapamycin (RAPA, an autophagy activator that acts via inhibiting the mTORC1 pathway) [19,21,22,23] administration, moreover, was able to partially restore the autophagy marker levels.

Clearly, in KD, restoring the presence of the functioning enzyme in the brain is mandatory. However, brain targeting in enzyme replacement therapy did not lead to a long-lasting result either in mice [24,25] or human patients [26,27], who died from further complications. It is therefore essential to discover and understand all the molecular mechanisms that lead to the pathological imbalances in KD, to treat this pathology with multiple approaches by acting on the affected pathways, rescuing the KD phenotype.

In specialized cells (e.g., fibroblasts), cell sensing and migration are achieved through focal adhesions (FAs) [28]. FAs are protein clusters, and their components assemble on the transmembrane integrins adhered to the substrate [29]. Later, FAs’ maturation and protein recruitment are guided by actomyosin tensile stress [30]. At the trailing edge, old and larger FAs are targeted by microtubules, dynamins and FAK that interact; FAs are hence disassembled and destined to an autophagic fate, and their components are recycled [31]. At this point, the cell has left the anchor at the trailing edge, represented by the old FAs, and can pull itself to the leading edge. The training movement is transmitted through cells thanks to the actin cytoskeleton via cell–cell junctions [32]. These junctions are mainly represented by cadherins, which are a family of transmembrane proteins mediating adhesion and movement transmission between two neighbouring cells. N-cadherin in particular have a role in migration [33].

Fibroblasts are a simple and accessible cell model easily collectable from both patients [34] and mice [35], and they represent the most common cells in the connective tissue. We can find them in the interstitial spaces of the tissues, where they synthesize and produce extracellular matrix (ECM) components. They can migrate and remodel the environment and signal to other cells their fate and directions [36]. They have a role in injury healing and inflammation regulation, thanks to their ability to produce and release chemokines [37]. Therefore, migration and interaction with matrices and other cells are fundamental for fibroblasts’ activities.

Here, we studied mechanotransduction and migration processes in primary fibroblasts collected from twitcher mice. Via time-lapse microscopy, we investigated single-cell migration and wound healing in cell monolayers. We evaluated the development and the expression of FA intracellular components, and also that of N-cadherin junctions, via immunocytochemistry and Western blot. We investigated the cross-talk between the mechanotransduction and autophagy processes by looking for the presence and localization of the neighbour of BRCA1 gene 1 protein (NBR1) [38,39], the protein needed for the selective autophagy, recycling, and turnover of FAs. Furthermore, we tested whether RAPA treatment would rescue the impaired twitcher fibroblasts’ responses.

## 2. Methods

### 2.1. Animals and Cells

Twitcher heterozygous mice (GALC^+/−^ C57BL/6J mice; Jackson Laboratory, Bar Harbor, ME, USA), a model of GALC deficiency/Krabbe disease kindly donated by Dr. A. Biffi and Dr. A Gritti (San Raffaele Telethon Institute for Gene Therapy, Milan, Italy), were used as breeder pairs to generate homozygous (GALC^−/−^, abbreviated elsewhere as HOM) and wild-type (GALC^+/+^, abbreviated as WT) mice. Animals were maintained under standard housing conditions and used according to the protocols and ethical guidelines approved by the Ministry of Health (permit number: CBS-not. 0517; approved 4 January 2018). For genotyping purpose, mice’s genomic DNA was extracted from clipped tails using proteinase K digestion and subsequent genomic DNA extraction (Tissue-DNA Mini Kit, Euroclone, Pero, Italy), as previously detailed [12]. The genetic status of each mouse was later determined from the genome analysis of the GALC mutation, as reported by Sakai and colleagues [40].

### 2.2. Cell Culture, Drugs’ Treatments, and Cell Viability Assay

Adult mouse fibroblast cultures were obtained from WT and HOM twitcher adult mice (P20–P24). We adapted the protocol established in the laboratory of Dr. Evan Eichler (University of Washington Department of Genome Sciences) as follows. Mice were sacrificed and ears were extracted, washed with sterile water, and cut into small pieces. Pieces were collected in a 1.5 mL microcentrifuge tube and 0.5 mL of collagenase XI (C7657, Sigma Aldrich, Milwaukee, WI, USA) diluted in high-glucose Dulbecco’s Modified Eagle Medium (DMEM; 3105304, Thermo Fisher, Waltham, MA, USA) (approximately 600 CDU per mouse). After 2 h of incubation at 37 °C, samples were centrifuged for 5 min at 200× *g*, and the supernatant was carefully discarded. Pellet was washed with 1.5 mL of sterile phosphate-buffered saline (PBS; D8537-500 mL, Sigma Aldrich), resuspended, and centrifuged as above, discarding the supernatant. Then, 0.5 mL of 0.05% Trypsin-EDTA (Cat# 25300-054, Thermo Fisher) was added for 30 min at 37 °C. Finally, the pellet was resuspended in complete DMEM (high-glucose DMEM supplemented with 10% of heat-inactivated foetal bovine serum (FBS; Cat#16000-044, Thermo Fisher), 4 mM-glutamine, 1% MEM Nonessential Amino Acids (Cat#11140-050, Thermo Fisher), and 1% penicillin–streptomycin (Cat#15140-122, Thermo Fisher). The suspension was pipetted up and down in a Pasteur pipette and plated. Cells were incubated in a 37 °C, 5% CO_2_ humidified atmosphere tissue culture incubator. Cells were cultured on standard cell plates for tissue culture (without any additional coating) and used for experiments within the 10th passage in vitro.

For cell treatments, fibroblasts were treated with Rapamycin 100 nM (RAPA; CAS 53123-88-9, InvivoGen, San Diego, CA, USA) and/or Bafilomycin 200 nM (BAF; B1793, Sigma Aldrich). RAPA and BAF were dissolved in dimethylsulfoxide (DMSO; CAS#67-68-5, Sigma Aldrich), at 10 mM and 0.2 mM, respectively. As in [19], RAPA was used at a concentration of 100 nM in DMEM medium for 24 h (RAPA) to stimulate autophagy. BAF was administrated at a concentration of 200 nM for 4 h, and added before the end of RAPA treatment (RAPA+BAF to block autophagy. Control experiments were conducted using the same quantity of DMSO solvent present in RAPA and BAF treatment, which never exceeded 0.6% *v*/*v*. For the experiments, cells were plated 24 h before the treatments.

RealTime-Glo™ MT Cell Viability Assay (G9712, Promega, Fitchburg, WI, USA) was used for cell proliferation and viability measurements. The assay is based on the reduction in NanoLuc^®^ substrate from metabolically active cells. In a 96-well plate, we plated 4000 cell/well and followed the assay instructions (Protocol for Continuous-Read Format). Luminescence was read with the plate reader GloMax DISCOVER (Promega). We performed each sample in triplicate in the assay, for a total of *n* = 3 experimental replicates.

### 2.3. Single-Cell Migration

WT and HOM fibroblasts were seeded at a density of 1.5 × 10^4^ cells/cm^2^, and, the day after (24 ± 1 h), live-imaged for 24 h, every 15 min, with an inverted Nikon-*Ti* wide-field microscope (Nikon, Tokyo, Japan) equipped with perfect focus system and cell incubator (Okolab, Naples, Italy), with an air 20× (NA 0.45, Plan-Fluor, Irving, TX, USA) objective, and a CCD ORCA R2 (Hamamatsu, Iwata City, Japan).

Cell movies were analysed with the ImageJ manual tracking plugin MTrack as stated by Tonazzini and colleagues [41]. We measured (i) the *average speed* (V, the mean displacement achieved in 1 h; in μm/h), (ii) the *total displacement* (the total distance covered by each cell from *t* = 0 to *t* = 24 h; in μm).

We analysed at least 10 cells for each sample, and we performed *n* = 4 independent experiments for each condition.

### 2.4. Wound Healing

WT and HOM fibroblasts were seeded at a density of 45,000 cells/cm^2^ (1.0 × 10^4^ cells/well chamber) in the Ibidi Culture-Insert 2 Well for wound healing assay (81176, IBIDI GMBH, Gräfelfing, Germany) in a 6-well dish, and processed as previously reported [42]. The next day (after 24 h from seeding) the Ibidi Culture-Inserts were removed (*t = 0*) and cells were live-imaged for 48 h, every 15 min, in a time-lapse created using a Nikon-*Ti* wide-field microscope and 20× objective. For experiments in treated conditions, after the 24 h from seeding, cells were treated with RAPA: the inserts were removed after 14 h from RAPA administration (to perform the migration experiment across the 24 h RAPA treatment condition) and from this point (*t = 0*) cells were live-imaged for 48 h, as before. The two monolayers were separated by an empty gap with an averaged width of 497 ± 20 µm (mean ± SD, *n* = 18) at *t* = 0. The wound area was tracked as an ROI (delimited by the monolayer’s borders and the image borders) and measured at *t* = 0, *t* = 24 (T24), and *t* = 48 (T48) and reported as the percentage (%) of area closure [43]. The number of scattered cells in the wound at 24 h was also quantified and reported as number of scattered cells/wound, as in [41].

We also tracked the movements of clusters of cells (i.e., the nuclei of 4–5 neighbouring cells) in the monolayer’s front, similarly to Li et al. [44]. These cell group tracks were identified using Mtrack, as described above for the single-cell experiments. From each track, the coordinates of the starting point X,Y was subtracted to begin from the origin of the axes.

For each condition, at least *n* = 3 independent experiments were performed.

### 2.5. Immunostaining

WT and HOM fibroblasts were cultured for 48 h (±2 h) in untreated/treated conditions, fixed for 10 min with 4% formaldehyde in PBS at room temperature (RT), and processed as previously reported [45]. Briefly, the primary antibody mix was composed of GDB buffer (0.2% BSA, 0.8 M NaCl, 0.5% Triton X-100, 30 mM phosphate buffer, pH 7.4), Alexa Fluor™ 647 Phalloidin (A22287, 1:40, Thermo Fisher) to stain the actin fibres (F-actin), and the following primary antibodies: anti-NBR1 (1:200; PA-30085, Thermo Fisher); anti-Vinculin (1:100; VINC; ab18058, Abcam, Cambridge, UK); anti-N-Cadherin (1:200; N-CAD; 610921, BD Transduction Laboratories, Franklin Lakes, NJ, USA); anti-YAP1 (1:200; ab39361, Abcam). Then, samples were incubated for 1 h at RT with the appropriate secondary antibody, Alexa Fluor 488 (anti-mouse; Cat#A21202) or Alexa Fluor 555 (anti-rabbit; Cat#A31570), diluted 1:100 in GDB buffer and mounted using Fluoroshield mounting medium with DAPI (F6057, Sigma Aldrich).

### 2.6. Confocal Fluorescence Microscopy

Immunostained cells images were collected with a laser scanning confocal microscope TCS SP2 (Leica Microsystems, Wetzlar, Germany) equipped with 40× oil objective, using UV, Ar, HeNe, and He lasers lines (for 405, 488, 561, and 633 nm excitation, respectively). Each reported confocal image was obtained from a z-series (stack depth was around 10–15 μm, with steps of 1–1.5 μm), with each image averaged three times. The resulting z-stack was processed in ImageJ (Ver. 1.53c, NIH USA) into a single image using the “*Z-project*” and “*Max intensity*” options. The confocal settings were kept the same for all the scans when the fluorescence intensity was compared.

### 2.7. FA, NBR1, N-CAD, and YAP Image Analysis

For the analysis of FAs (on VINC images), N-CAD, and NBR1 signals, every image was thresholded using the “Threshold” tool of ImageJ (NIH, USA) (with a threshold of 110 for the NBR1 aggregates, 70 for N-CAD junctions, and 50 for VINC+ FAs) [19,46]. The area covered by each cell was manually selected as a region of interest (ROI) on bright-field images (merged with the DAPI and the phalloidin signals). The ROIs have been applied to the correspondent binary images for the analysis of NBR1, N-CAD, or VINC signal in each cell. Here, the number of NBR1, N-CAD, or FA clusters (VINC signals) was analysed using the “Analyse Particles” plugin in ImageJ by setting “Size (Micron^2^)” = from 0.1 to Infinity and “Circularity” = from 0.10 to 1.00. We analysed all the cells present and completely visible in each image. For the analysis of NBR1 spots, we reported the mean number of spots/cell.

For the analysis of N-CAD signal at the cell borders, an ROI was designed across the contact perimeter between every two cells. We kept a comparable perimeter between pairs of different cells, with a length of approximately 25–30 μm and with a depth of the perimeter for the ROI of 5–10 μm in cells.

The YAP1 signal was quantified by drawing both the cell and the nucleus ROIs for each cell [47]. The YAP1 intensity was measured using the ImageJ “Measure” tool “*Mean grey value”* and reported as the nuclear/cell ratio.

For each condition, we performed at least n = 3 independent experiments, and a minimum number of 15 cells per sample were evaluated.

### 2.8. Western Blot

WT and HOM fibroblasts were cultured on standard plates until subconfluence and then lysed on ice in RIPA buffer (R0278; Sigma Aldrich) containing a protease and phosphatase inhibitor cocktail (Complete and PhosSTOP, Roche Diagnostics, Basel, Switzerland). Cell lysates were centrifuged (15,000× *g* for 25 min, 4 °C), and then the supernatants were tested for protein concentration with a protein assay kit (Micro BCA™, Thermo Scientific Pierce). The samples were mixed with Laemmli buffer containing β-mercapto-ethanol, boiled for 5 min, and used for gel electrophoresis [45]. Briefly, lysates (20 µg/lane) were resolved via gel electrophoresis (SDS-PAGE) using Gel Criterion XT-Precast polyacrylamide gel 4–12% Bis-Tris (Biorad, Hercules, CA, USA), transferred to nitrocellulose membranes, and probed overnight at 4 °C with primary antibodies: FAK (1:1000; ab40794, Abcam); phospho(Tyr397)-FAK (1:1000; ab4803, Abcam); paxillin (1:5000; ab32084, Abcam); p-paxillin (1:1000; ab75740, Abcam); GAPDH (1:3000; G8795, Sigma Aldrich). Membranes, after incubation with the appropriate peroxidase-linked secondary antibodies (goat anti-Rabbit/Mouse IgG-HRP Conjugate, Biorad; 1:2500), were developed by ClarityTM (170-5060, Biorad) enhanced chemiluminescent (ECL) substrates; the SuperSignal West Femto (#34095, Thermo Fisher) system was used to develop signals from phosphorylated antibody phospho-FAK and phospho-PAX. The chemiluminescent signal was acquired using an ImageQUANT LAS400 scanner (GE Healthcare Life Sciences, Uppsala, Sweden), and proteins’ bands were quantified using ImageJ. The results were normalized to the respective total GAPDH content. We ran at least n = 3 independent samples for each condition.

### 2.9. Statistical Analysis

Data are reported as average values ± the standard error of the mean (mean ± SEM), unless indicated otherwise. All cell experiments were repeated at least three times independently for each condition (n ≥ 3). Data were statistically analysed using the GraphPad PRISM 5.00 program (GraphPad Software, San Diego, CA, USA). If not indicated otherwise, Student’s *t*-test (unpaired, two-tailed) was used to compare WT and HOM cells. One-way ANOVA Bonferroni’s multiple comparison test was used for comparison between untreated/ treated conditions. Statistical significance refers to *p* < 0.05.

## 3. Results

### 3.1. GALC-Deficient Fibroblasts Showed Impaired Migration at the Single-Cell Level and in Monolayer Wound Healing

To evaluate if GALC deficiency could affect cell motility, we performed single-cell and wound-healing migration experiments on WT and HOM primary fibroblasts established from twitcher mice.

**Figure 1:**
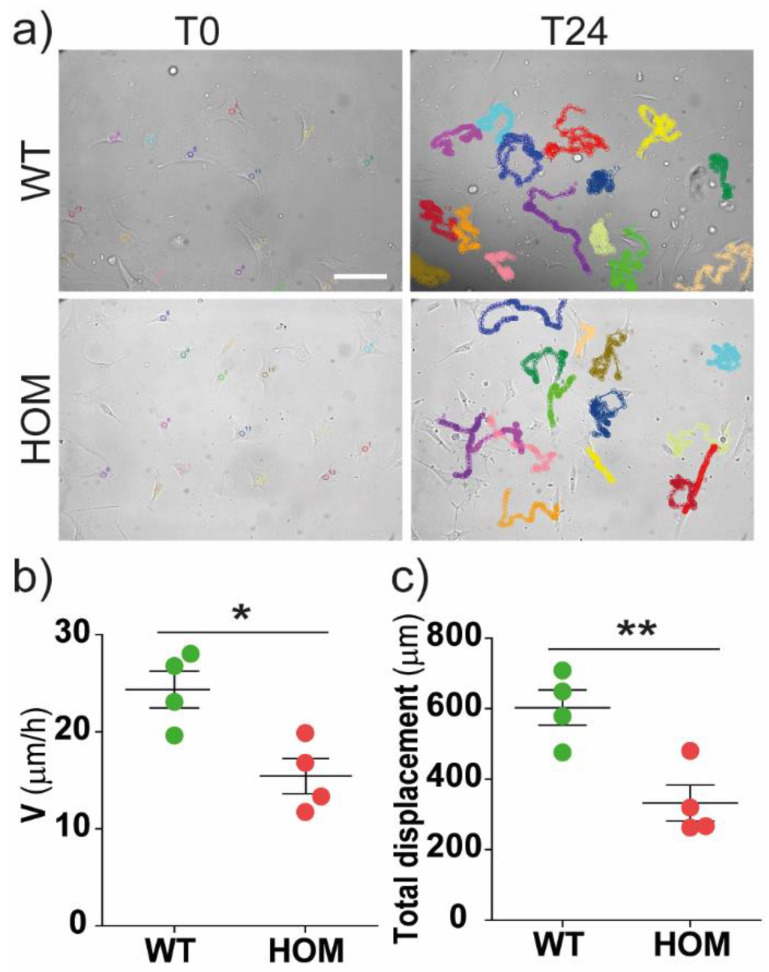
Single-cell migration. (**a**) Representative bright-field images of WT and HOM fibroblast tracks (*coloured trajectories*) during 24 h live-imaging migration experiment. Scale bar = 100 µm. (**b**) Mean speed migration (μm/h) of WT and HOM fibroblast cells. (**c**) Total cell displacement over *t* = 24 h for WT and HOM cells. */** *p* < 0.05/0.01 WT vs. HOM, Student’s *t*-test, unpaired. Data = mean ± SEM, *n* = 4.

At first, fibroblast cells were plated at a low concentration, and were able to move freely and independently. Single-cell migrations were tracked via live-cell imaging for 24 h (Figure 1a) and analysed. The mean distance covered in every 15 min step by WT cells was greater than that for HOM cells, leading to a greater average cell migration speed in WT cells than HOM cells (24.4 ± 1.9 and 15.3 ± 1.8 μm/h, respectively; *p* < 0.05 WT vs. HOM) (Figure 1b). Accordingly, the total cell displacement over 24 h for WT cells was 1.8 times greater than for HOM cells (602.9 ± 50.0 and 332.6 ± 51 μm, respectively; *p* < 0.01 WT vs. HOM) (Figure 1c).

**Figure 2:**
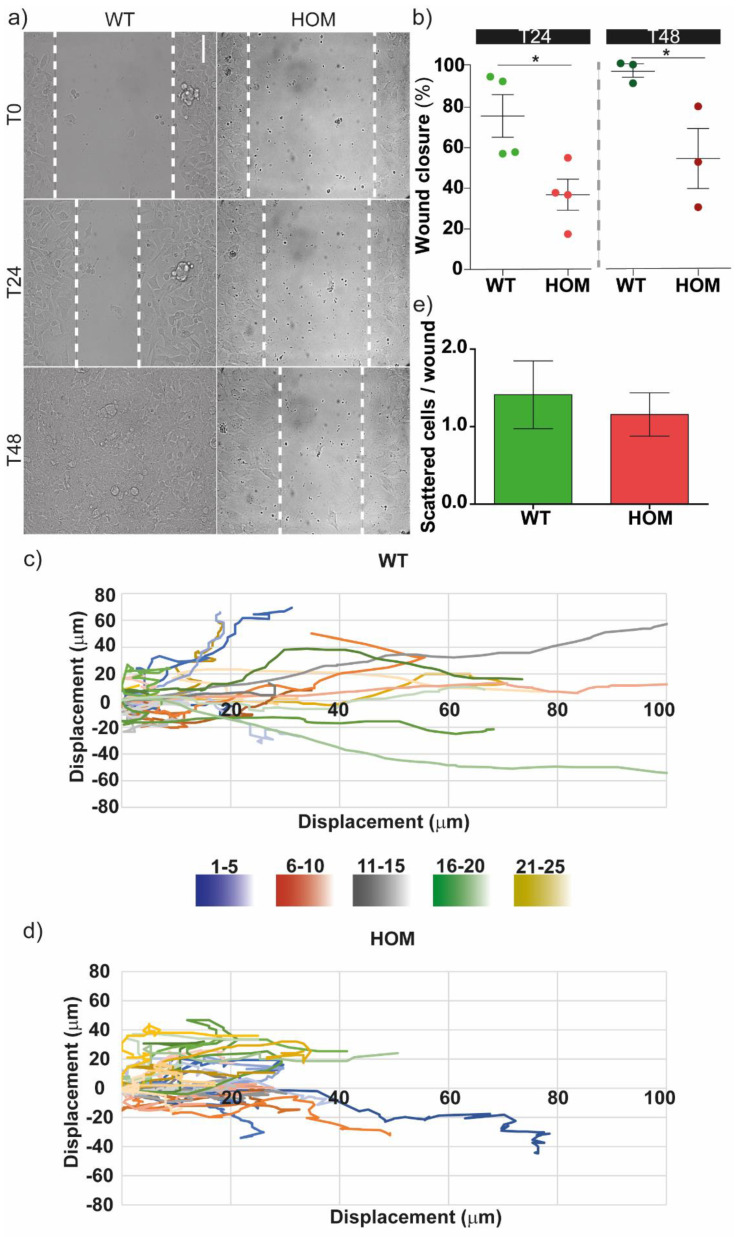
Cell migration during wound healing. (**a**) Representative bright-field images of WT and HOM fibroblasts’ monolayers at *t* = 0–24–48 h. Scale bar = 100 µm. (**b**) Wound closure (%) at *t* = 24 h (*left*, lighter colours) and *t* = 48 h (*right*, darker colours). * *p* < 0.05 WT vs. HOM, Student’s t-test, unpaired. Data = mean ± SEM, *n* = 4 at *t* = 24 h, n = 3 at *t* = 48 h. (**c**,**d**) Representative cell tracks (for 24 h) of WT and HOM clusters of cells (i.e., the nuclei of 5 neighbouring cells for each cluster) at the monolayer’s front, represented with flower plots: each cell of a cluster has a different shade, and all the neighbouring cells from the same cluster (area) are shown with shades of the same colour. (**e**) Scattered cells in the wound at 24 h. Data = mean ± SEM, *n* = 4.

We then performed the wound healing assay, assessing the fibroblasts’ monolayer front migration, where cell–cell interactions and directionality play a fundamental role. Cells, plated in a monolayer separated by a gap of approximately 500 µm, were tracked for 48 h (Figure 2a). In the first 24 h, WT cells covered around 70% of the gap, while at 48 h, the wound was completely closed (Figure 2b). HOM cells showed delayed wound closure compared to WT cells at both time points (*p* < 0.05 WT vs. HOM); here at 48 h, HOM cells were not able to close the gap (Figure 2b). We further analysed our time-lapse video of wound healing experiments by tracking clusters of cells (i.e., the nuclei of 4–5 neighbouring cells, in a restricted region of the monolayer’s front) for 24 h (Figure 2c,d). Both WT and HOM fibroblasts showed a group migration directed to the wound-free area; cells from the same cluster often migrate together and in similar directions. WT cells showed longer tracks, in agreement with their faster migration and faster wound closure. We quantified the scattered cells in the wound area beyond the monolayers’ edges after 24 h (Figure 2e). The number of these scattered cells in the wound did not vary between WT and HOM cells, suggesting that the monolayer behaviour is similar.

We then checked if GALC deficiency impacted fibroblasts’ proliferation, which in turn might affect the HOM wound-healing migration performance. Cell proliferation was similar between WT and HOM cells at different time points (Figure S1a). Finally, we performed single-cell migration and wound healing experiments, also in low-serum conditions (i.e., 0.2% FBS), to further demonstrate the null contribution of cell proliferation in the wound healing response. Both WT and HOM fibroblasts presented a general slowdown compared to their performance in complete medium (i.e., 10% FBS), and HOM cells always showed the same trend, with worse migration performances than WT cells (Figure S1b–d).

Overall, these data highlight reduced motility in GALC-deficient fibroblast cells, both at the single-cell level and during wound healing.

### 3.2. The Number of Focal Adhesions Increases in GALC-Deficient Fibroblasts

**Figure 3:**
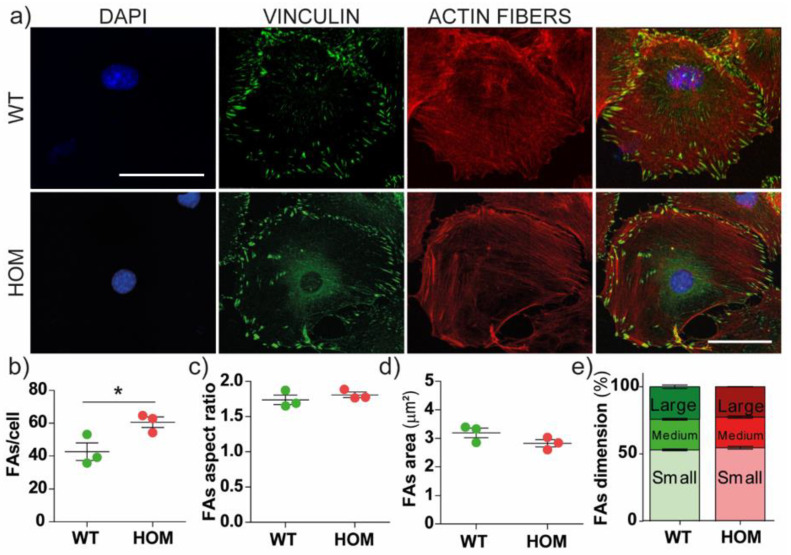
Focal adhesions in primary twitcher fibroblasts. (**a**) Representative confocal images of WT and HOM fibroblasts immunostained for nuclei (*blue*), vinculin (*green*), actin (*red*); scale bar = 50 µm. (**b**–**e**) FA analysis: FAs’ (**b**) number per cell, (**c**) aspect ratio, (**d**) mean area (μm^2^). (**e**) Distribution of FA size: the % of small (area ≤ 3 µm^2^), intermediate (3 < area ≤ 6 µm^2^), and large (area > 6 µm^2^) FAs is reported for WT and HOM cells. * *p* < 0.05 WT vs. HOM, Student’s *t*-test, unpaired. Data = mean ± SEM, *n* = 3.

Because it is well known that the migration process is mediated by focal adhesions (FAs), we investigated FA development to determine if GALC deficiency could affect the mechanotransduction process. We immunostained cells against vinculin (VINC), a protein of FA clusters (Figure 3a). We also stained cells with phalloidin to visualize the actin fibres. HOM fibroblasts showed an increased number of VINC-positive FAs per cell compared to WT (WT = 43 ± 5, HOM = 61 ± 3; *p* = 0.05 WT vs. HOM; Figure 3b). We report that the mean cell size was similar between WT and HOM cells (3078 ± 562 and 3200 ± 364 μm^2^, respectively). We then analysed FAs’ morphology (i.e., their aspect ratio, the ratio between the longest and the shortest axes of each FA) and maturation (i.e., FA area). The shaping and size of FAs were not different between WT and HOM fibroblasts (Figure 3c–e). The maturation was also evaluated through FAs’ area distribution (Figure 3d), and FAs were sorted into three size categories (small (area ≤ 3 μm^2^), medium (3 μm^2^ < area ≤ 6 μm^2^), and large (area > 6 μm^2^)), again showing no differences in FA maturation (Figure 3e). We also quantified the cell spreading area (size) of WT and HOM fibroblasts, which did not show any differences (Figure S2a).

Finally, we measured the activation of the focal adhesions’ intracellular pathway at the molecular level, looking for the presence and activation of the proteins focal adhesion kinase (FAK) and paxillin (PAX). Western blot results do not show significant differences in FAK and PAX activation levels (Figure S2b–e).

Overall, data show that FAs were upregulated in HOM fibroblasts; the GALC deficiency affected the number of FAs, but not their morphology or maturation. Moreover, the intracellular activation of FAs’ pathway was unperturbed in HOM fibroblasts.

### 3.3. Dysregulated Distribution and Dimension of N-CAD Junctions

**Figure 4:**
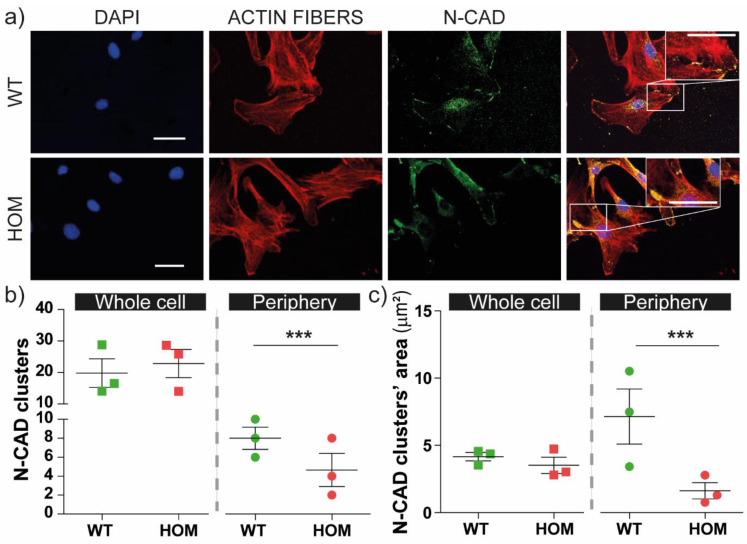
Cell–cell junctions in WT and HOM fibroblasts. (**a**) Representative confocal images of WT and HOM fibroblasts immunostained for (from left to right) nuclei (*blue*), actin fibres (*red*), N-CAD (*green*); scale bar: 25 μm. (**b**) Quantification of N-CAD clusters: number of N-CAD clusters per cell (*left side*, squares) and at cell periphery (i.e., at the cell–cell borders, *right side*, dots). (**c**) Analysis of N-CAD clusters’ area in whole cell (*left side*, squares) and at the cell–cell periphery (*right side*, dots). *** *p* < 0.001 WT vs. HOM, Student’s *t*-test, unpaired. Data = mean ± SEM, *n* = 3.

It is known that FAs couple with intercellular junctions thanks to the cytoskeleton/actomyosin compartment. We studied cell–cell junctions and in particular the N-cadherins (N-CAD), which are important for migration in WT and HOM fibroblasts (Figure 4a).

We evaluated the number of N-CAD clusters (Figure 4b) and their area (in μm^2^; Figure 4c) in the whole cell and at the cell–cell borders. In the whole cell, the number/cell and the mean area size of N-CAD clusters were similar between WT and HOM fibroblasts (Figure 4b–c, *left panels*). Interestingly, when we focused on the N-CAD clusters present at the cell–cell borders (i.e., at the cell periphery contacting other cells), we found that N-CAD clusters were significantly less numerous and smaller in HOM cells than WT cells (*p* < 0.001 WT vs. HOM; Figure 4b,c).

These results indicate that HOM fibroblasts present a reduced number of N-CAD junctions between neighbouring cells compared to WT, suggesting the low strength of cell–cell junctions and likely the wound healing impairment.

### 3.4. YAP Mechanosensing Activation

YAP-TAZ signalling acts as a sensor for cytoskeletal tension and regulates both cell mechanotransduction [48,49] and motility.

**Figure 5:**
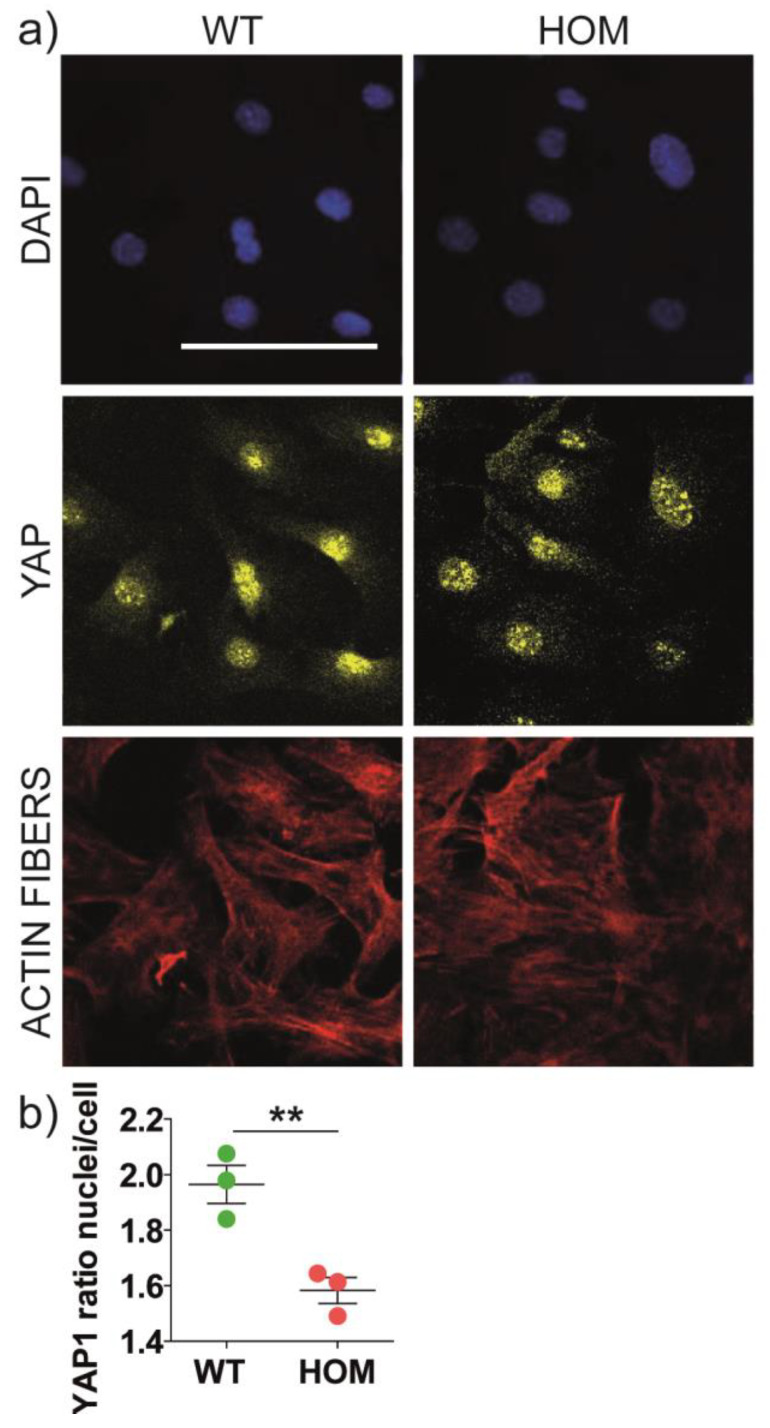
YAP localization and activation in primary twitcher fibroblasts. (**a**) Representative confocal images of WT and HOM fibroblasts immunostained for (from top to bottom) nuclei (*blue*), YAP1 (*yellow*), actin fibres (*red*); scale bar: 50 μm. (**b**) Quantification of YAP1 expression ratio between nuclei and cell soma. ** *p* < 0.01 WT vs. HOM, Student’s *t*-test, unpaired. Data = mean ± SEM, *n* = 3.

We plated cells on glass coverslips, an almost rigid substrate, and investigated YAP activation (i.e., the ratio between its nuclear and cytoplasmic localization) [50] via immunocytochemistry (Figure 5a). We found that the mean YAP ratio was significantly lower in HOM fibroblasts compared to WT fibroblasts (WT ratio = 1.97 ± 0.07, HOM ratio = 1.58 ± 0.05; *p* < 0.01 WT vs. HOM) (Figure 5b), suggesting a reduced activation of this mechanosensitive pathway in HOM fibroblasts.

### 3.5. Autophagy Activation Rescues Cell Migration

**Figure 6:**
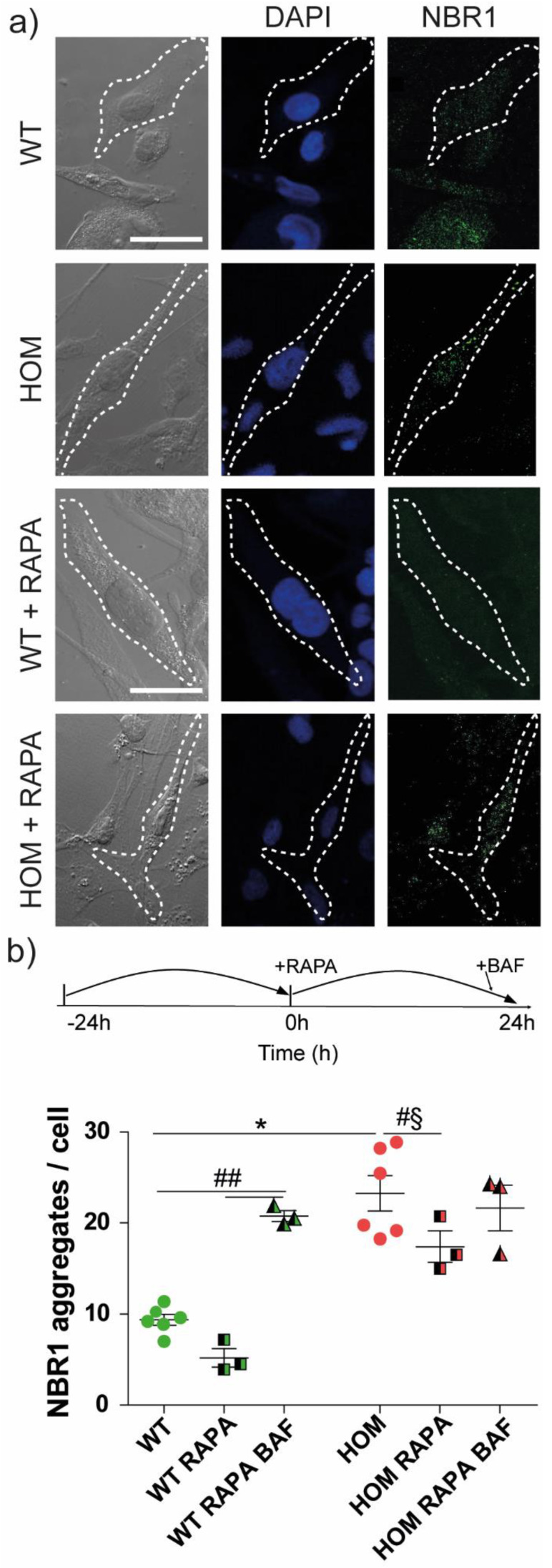
NBR1 analysis in WT and HOM primary cells. (**a**) Representative confocal images of twitcher fibroblasts, in control conditions (untreated (UT) and vehicle-treated (DMSO)) and treated with RAPA (from left to right): bright-field nuclei (*blue*), NBR1 (*green*); scale bar: 50 μm. The white dotted lines highlight an example of cell contour, for a better visualization. (**b**) The treatment scheme over time (inset): cells were treated for 24 h with 100 nM RAPA, one day after seeding, and then treated with BAF (*grey*) for a few hours, if required. Quantification of NBR1 aggregates in control cells (untreated (UT) and vehicle-treated (DMSO); dots), in RAPA-treated cells (half-coloured squares), and in RAPA + BAF-treated cells (half-coloured triangles). * *p* < 0.05 WT vs. HOM, Student’s *t*-test, unpaired. ^##^ *p* < 0.01, one-way ANOVA, Bonferroni’s test within each genotype; ^#§^ *p* < 0.05, Bonferroni’s selected test. Data = mean ± SEM, *n* ≥ 3.

We investigated the presence and localization of NBR1, a protein needed for FA turnover and recycling. We evaluated the presence of NBR1 aggregates in WT and HOM cells (Figure 6a, *top*). Importantly, HOM fibroblasts showed a greater number of NBR1 aggregates per cell than WT fibroblasts (WT = 9.4 ± 0.6, HOM = 23 ± 2; *p* < 0.001 WT vs. HOM; Figure 6b).

We then treated twitcher fibroblasts with the autophagy activator RAPA for 24 h in order to determine if autophagy activation regulates the number of NBR1 aggregates (Figure 6a, *bottom*), as previously discovered [19]. To establish a control, we treated cells with DMSO (RAPA vehicle) alone. DMSO had no effects either on WT or HOM cells, as expected; therefore, DMSO data were pooled altogether with untreated (UT) cells (WT UT = 8.6 ± 0.8, WT + DMSO = 10 ± 0.7; HOM UT = 22 ± 3, HOM + DMSO = 24 ± 3) and used as the control condition.

First, RAPA induced a clearance process in WT cells by reducing the number of NBR1 aggregates (WT + RAPA = 5 ± 1; *p* < 0.01 WT vs. WT + RAPA, Bonferroni’s test) (Figure 6b). RAPA induced a small decrease in the number of NBR1 aggregates in HOM cells too (HOM + RAPA = 17 ± 2; *p* < 0.05 HOM vs. HOM + RAPA, Bonferroni’s selected test; Figure 6b).

Then, to understand if a block in the autophagic flux could inversely impact the presence of NBR1 aggregates in HOM fibroblasts, we administered bafilomycin A1 (BAF) after RAPA (Figure 6b). BAF inhibits the vacuolar pump H^+^ ATPase, preventing the acidification of lysosomal vesicles and disrupting the autophagosome fusion with lysosomes, thus blocking the autophagy process [19] (Figure S3a). We observed an increase in the NBR1 aggregates’ number per cell in WT fibroblasts treated with BAF (WT + RAPA + BAF = 20.8 ± 0.6; *p* < 0.001 WT and WT + RAPA vs. WT + RAPA + BAF, Bonferroni’s test) (Figure 6b). The number of NBR1 aggregates in HOM cells treated with BAF recovered to the untreated condition level (HOM + RAPA + BAF = 21.7 ± 2.5).

These data indicate that RAPA administration reduced the presence of NBR1 aggregates both in WT and HOM fibroblasts, likely via autophagy activation. The BAF treatment (i.e., blocking autophagy) induced an almost opposite effect. In WT fibroblasts, BAF administration increased the number of NBR1 aggregates, as expected, while in HOM fibroblasts, the NBR1 aggregates’ value just returned to the untreated level, without any further increase. These results suggest the presence of dysregulation in the autophagy process in HOM fibroblasts.

**Figure 7:**
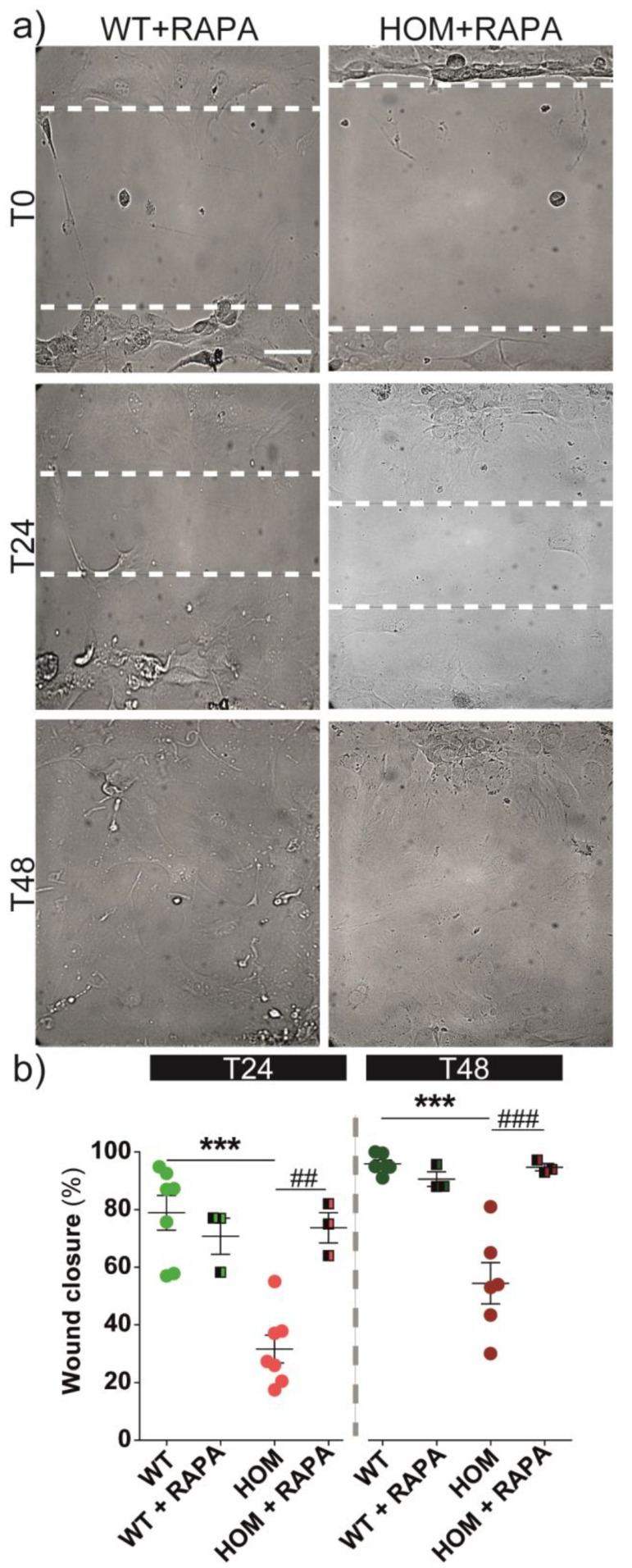
Cell migration during wound healing after RAPA-induced autophagy activation. WT and HOM fibroblasts were treated without or with 100 nM RAPA (14 h before t = 0). (**a**) Representative bright-field images of WT and HOM fibroblasts’ monolayers at *t* = 0-24-48 h. Scale bar = 100 µm. (**b**) Wound closure (%) at *t* = 24 h (*left*, lighter colours) and *t* = 48 h (*right*, darker colours). *** *p* < 0.001 WT vs. HOM, Student’s *t*-test, unpaired. ^##/###^ *p* < 0.01/0.001, one-way ANOVA, Bonferroni’s test. Data = mean ± SEM, *n* ≥ 3.

In order to understand if autophagy could influence the migration behaviour of fibroblasts, we tracked (Figure 7a) and measured (Figure 7b) cell group migration in the presence of RAPA. RAPA was administered 14 h before the wound healing experiment (i.e., to perform the migration experiment across the 24 h RAPA treatment condition). Again, DMSO solvent per se had no effects on wound closure, and again these two conditions (UT and DMSO) were pooled together (0.8 ± 0.1 and 0.8 ± 0.04, WT UT and WT + DMSO, respectively; 0.37 ± 0.08 and 0.25 ± 0.02 HOM UT and HOM + DMSO, respectively). RAPA treatment did not affect the wound closure performances in WT cells (Figure 7b). On the other hand, HOM fibroblasts treated with RAPA had an improved migration rate, thus showing a full rescue of the cell group migration (at T24 *p* < 0.01 HOM vs. HOM + RAPA; at T48, *p* < 0.001 HOM vs. HOM + RAPA, respectively; Bonferroni’s test), and after 48 h, HOM + RAPA cells were able to close the wound gap as WT fibroblasts did. As a further control, we checked the effects of RAPA on WT and HOM fibroblasts’ proliferation: RAPA treatment did not affect cell viability or proliferation in either fibroblast (Figure S3b). In parallel with previous experiments, we also treated cells with RAPA + BAF. However, the BAF treatment completely blocked fibroblasts’ migration: both WT and HOM cells remained almost stacked without moving (Figure S3c), excluding the possibility of studying their migration.

**Figure 8:**
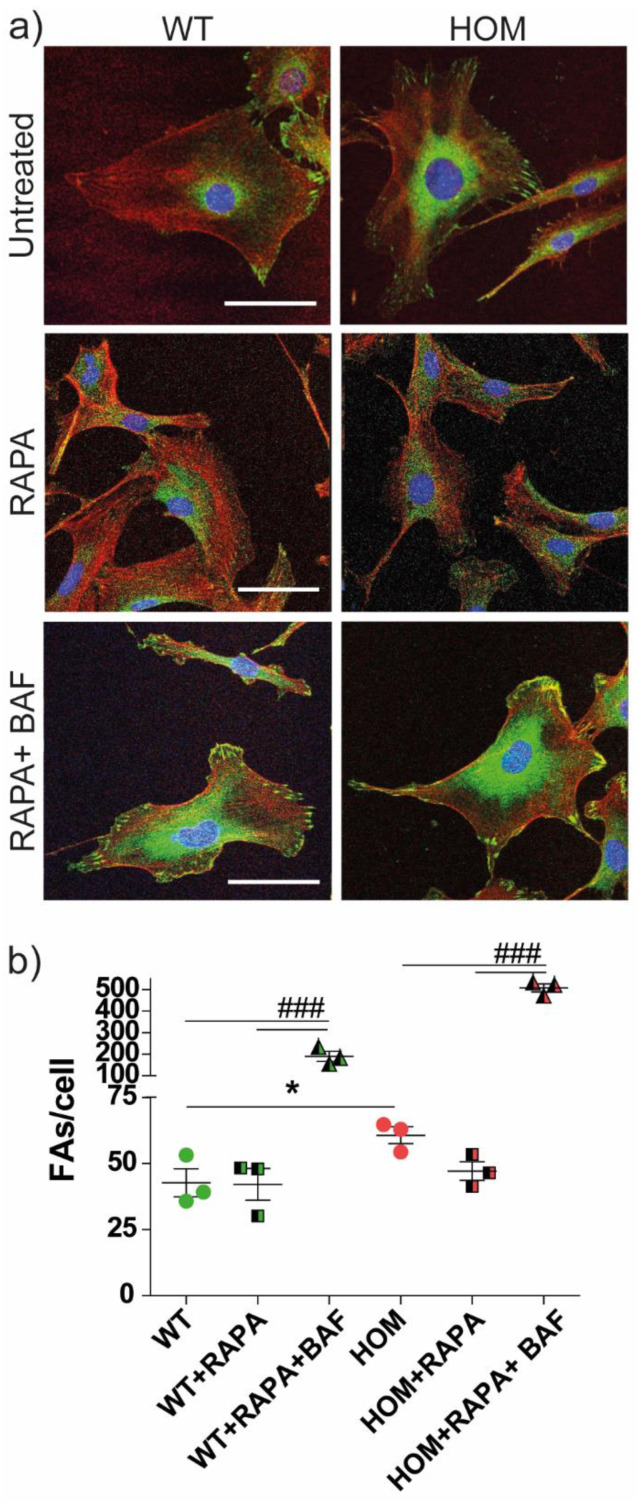
Focal adhesions in primary twitcher fibroblasts after RAPA treatment. (**a**) Representative confocal images of WT and HOM fibroblasts untreated and treated with RAPA and RAPA + BAF and immunostained for nuclei (*blue*), vinculin (*green*), actin (*red*); scale bar = 50 µm. (**b**) number of FAs per cell: * *p* < 0.05 WT vs. HOM, Student *t*-test, unpaired. Data = mean ± SEM, *n* = 3. ^###^ *p* < 0.001, one-way ANOVA, Bonferroni’s test within each genotype. Data = mean ± SEM, *n* = 3.

Because the RAPA treatment was able to fully rescue the migration deficits of twitcher fibroblasts and partially rescue the NBR1 aggregates, we examined its effects on FAs (Figure 8). At first, the treatment with RAPA did not affect the development or turnover of FAs in WT cells (WT = 43 ± 5, WT + RAPA = 42 ± 6; Figure 8b). In HOM fibroblasts, the RAPA treatment induced a slight reduction in the number of FAs (HOM = 61 ± 3, HOM + RAPA = 47 ± 4), which was only significant in a direct comparison between untreated and RAPA-treated conditions (*p* < 0.05, Bonferroni’s selected test). However, in HOM fibroblasts, RAPA brought the number of FAs/cell back to the basal WT level. The treatment with BAF after RAPA induced a significant increase in the number of FAs, in particular in HOM cells (WT + RAPA + BAF = 190 ± 23 and HOM + RAPA + BAF = 507 ± 19; *p* < 0.001 WT vs. WT + RAPA + BAF and HOM vs. HOM + RAPA + BAF; Bonferroni’s test).

Overall, these experiments indicate that RAPA treatment improves cell migration in GALC-deficient cells and this effect is mainly mediated by the reduction in FAs in HOM fibroblasts. RAPA induces a reduction in NBR1 aggregates and increases FA turnover.

## 4. Discussion

Here, we investigated the migration and adhesion processes in primary fibroblasts extracted from the twitcher mouse, a spontaneous KD model. We show that mechanotransduction is impaired in HOM fibroblasts, which lack the functional GALC enzyme and do not accumulate PSY. Both in single-cell migration and wound healing, we observed reduced motility in HOM cells compared to WT cells. In line with this, the number of FAs per cell was higher in HOM fibroblasts, while the number of N-CAD cell junctions was reduced at the cell periphery and YAP showed diminished nuclear translocation (i.e., activation). We also investigated the involvement of the autophagic process in this framework. We found an accumulation of NBR1 aggregates in HOM cells. Here, the treatment with the autophagy activator RAPA rescued the cell migration behaviour in HOM fibroblasts and slightly reduced the number of FA and NBR1 aggregates in HOM cells. Overall, we propose that GALC deficiency impacts HOM fibroblast cell mechanotransduction processes (i.e., FAs accumulation, impaired migration behaviour) via the impairment of the autophagic process.

Fibroblasts are an optimal cell model for studying how cells migrate and interact with the extracellular environment, and they do not accumulate PSY. We know that the migration process is regulated through FAs [51]. We found an increase in the number of FAs together with a slower migration rate in HOM fibroblasts compared with WT fibroblasts. However, FA morphology and maturation, together with FA intracellular pathway activation (at the level of FAK and PAX), were similar between WT and HOM cells. In agreement with our findings, there are several reports in the literature wherein an increased number of FAs is linked to a reduced cell migration rate [28,52,53,54].

FAs and N-CAD junctions are both mechanotransduction effectors and transducers, which mediate migration and can influence each other. In line with the increase in FAs in HOM cells, we observed a reduced presence of N-CAD-positive cell–cell junctions at the cell periphery, suggesting a molecular explanation for the reduced wound healing response [33,55].

YAP and YAP/TAZ signalling represent good molecular connectors, influencing cell migration [54,56]. It has been reported that YAP activation (i.e., translocation into the nucleus) determines an increase in the N-CAD protein level and FA formation and stabilization in primary glioma cell lines [50]. In line with this, YAP downregulation was linked to an increased number of FAs and delayed motility [54]. Here, we also show that HOM fibroblasts have reduced YAP nuclear translocation compared to WT fibroblasts. In our view, the reduced YAP activation could be linked to the increase in FAs, even if the contribution of other molecular pathways cannot be excluded and the direction of the reciprocal influence is not given [56]. In conclusion, HOM fibroblasts show several defects in mechanotransduction effectors.

Our previous work assessed the presence of dysregulations in the autophagy process in HOM models: we found an accumulation of LC3 puncta and increased LC3-II protein levels in MO3.13 cells treated with PSY [57], together with increased p62 levels in HOM fibroblasts in vitro, and in twitcher mouse brain in vivo [19]. Moreover, we showed that the treatment with the autophagy activator RAPA partially restores the autophagy markers levels and, in particular, the number of p62 aggregates in twitcher fibroblasts [19]. Our present results confirm the presence of dysregulations in the autophagy process in HOM fibroblasts.

The rate of cell migration is regulated by the turnover of FAs [51,58], and their disassembly is dynamically tuned by autophagy. The selective autophagy mediator for FAs is the autophagy cargo receptor NBR1 [39], which is analogous to the most famous p62 [59]. Kenific et al. showed that NBR1 depletion significantly inhibits cell migration [60,61]. We found an increase in the number of NBR1 aggregates in HOM cells, in accordance with what we found for p62 [19]. The treatment with RAPA (i.e., autophagy activation) led to a slight decrease in the accumulation of NBR1 aggregates, rescued the FA levels in HOM fibroblasts by reducing their number at the WT level, and fully rescued their migration behaviour. In line with these results, BAF treatment, which inhibits autophagosome–lysosome fusion and induces the block of autophagy [62,63], had an opposite effect, thus increasing NBR1 accumulation in WT cells and the number of FAs in both WT and HOM fibroblasts [64]. Unfortunately, BAF itself completely blocked the cell motion, both in WT and HOM, and we could not evaluate the migration after this treatment. Altogether, our results indicate that in HOM fibroblasts, RAPA treatment restores migration and induces the reduction in FA and NBR1 aggregates.

Previous studies showed that autophagy is important for the migratory phenotype [64,65]. We showed that autophagy congestion in HOM cells causes an increase in NBR1 and FA aggregates. This can cause a slowdown in cell migration. Recently, Weinstock et al. [66] showed that the ablation of GALC before P4 induced severe neurodevelopmental defects in the brainstem, demonstrating that temporal GALC expression is a major contributor to brainstem development. Cell migration is particularly important during neurodevelopment (e.g., in the cortical layers) [67], and here slight delays in migration can lead to important deficits. Therefore, our results indicate that: (i) the mechanotransduction and migration defects due to GALC deficiency and demonstrated in HOM fibroblasts can have a relevant role in neural cells; (ii) a boost to the autophagy process can help to counteract the cell migration deficits due to GALC deficiency and maybe other KD pathological features also.

Although additional studies are required to fully characterize the autophagy machinery in TWI mice, we suggest autophagy dysregulation as a new aspect involved in the molecular pathogenesis of KD: our data suggest that autophagy is dysregulated (but not impaired) in the TWI models [19,57]. Data overall suggest a higher basal level of autophagy activation in the missing GALC condition that is further stimulated in the presence of PSY. Specifically, GALC deficiency could lead to lysosomal dysfunction, partial blocking, and, consequently, saturating the autophagy flux. In line with this, altered autophagy and accumulation of lipids, especially cholesterol and glycosphingolipids, have been reported for a wide range of lysosomal storage disorders, including those without primary defects in glycosphingolipid or cholesterol degradation [68].

Recently, several findings have emerged in the literature concerning new aspects of KD pathogenesis that cannot be explained by PSY accumulation alone. Therefore, we studied mechanotransduction signalling in twitcher fibroblasts, which are a cell model that does not accumulate PSY and allows us to observe phenotypes related to PSY-independent pathological mechanisms. The measurement of this toxic lipid was addressed both in primary twitcher-mouse-derived fibroblasts [19] and in primary fibroblasts derived from KD patients [20], and both WT and HOM cells presented a similar level of PSY. To the best of our knowledge, only Li et al. [69] have reported a very modest accumulation of PSY levels in twitcher fibroblasts (i.e., PSY is 0.0010 arbitrary units in HOM mouse fibroblasts compared to 0.15–0.25 a.u. in the sciatic nerve and brain of HOM mice). We also addressed this problem and measured PSY in our WT and HOM fibroblasts, as in [19]: PSY levels were really low and almost undetectable (i.e., under the detection limit of the instrument, which is 18.2 pg/µg protein) in both our WT and HOM fibroblast samples (Figure S4). For this reason, we suggest that PSY is not primarily responsible for the adhesion and migration deficits that we observed in twitcher HOM fibroblasts. To better understand the role of GALC, further examinations have to be performed. Until now, research has focused mainly on PSY’s effects, due to its devastative impact on cells, but it is now emerging that GALC deficiency is pathological itself. Being able to discriminate the role of PSY from other GALC deficiency effects in this pathology is the first step to improve and design efficacy treatments.

To conclude, we propose that the mechanosensing impairment in HOM twitcher fibroblasts is due to the dysregulation of autophagy, which is primarily caused by GALC deficiency and leads to cell migration deficits. Our results suggest that a boost to the autophagy process can help to counteract some KD pathological features, laying the foundation for rapamycin preclinical testing.

## Data Availability

Not applicable.

## Supplementary Materials

The following supporting information can be downloaded at: https://www.mdpi.com/article/10.3390/biomedicines11030927/s1. Figure S1: Twitcher fibroblasts’ cell proliferation assay and migration in serum-starved conditions; Figure S2: FAs intracellular pathway in WT and HOM fibroblasts; Figure S3: Effects of different RAPA treatments on WT and HOM fibroblasts; Figure S4: Psychosine (PSY) content in HOM and WT primary fibroblasts [70,71].
