# Supplementary material for: Mechanotransduction Impairment in Primary Fibroblast Model of Krabbe Disease
Source: Biomedicines. 2023 Mar 16;11(3):927. doi: 10.3390/biomedicines11030927 (PMC10046230; doi:10.3390/biomedicines11030927)
Supplement: Supplementary file 1 [file biomedicines-11-00927-s001.zip › biomedicines-2243780-supplementary.pdf]

Supplementary section

Table of content

FigureS1…………………………………………………………………………………………………..1

FigureS2…………………………………………………………………………………………………..2

FigureS3…………………………………………………………………………………………………..3

FigureS4…………………………………………………………………………………………………..4

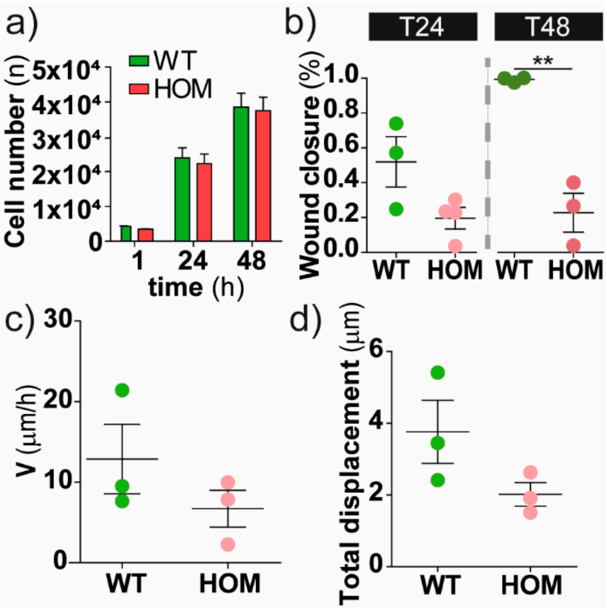

Figure S1. (a) Fibroblast cell proliferation assay: cells are reported as number (n) at different times (1 – 24 – 48 hours after seeding). b-d) twitcher fibroblasts' migration in serum-starved conditions (i.e. 0.2% serum). (b) Collective migration: Wound closure (%) at *t* = 24 h and *t* = 48 h. (c, d) Single-cell migration of WT and HOM fibroblasts: (c) mean speed migration (µm/h) and (d) total cell displacement (µm) after 24 h. ** P < 0.01 WT vs. HOM, Student's *t*-test, unpaired. Data = mean ± SEM, *n* ≥ 3.

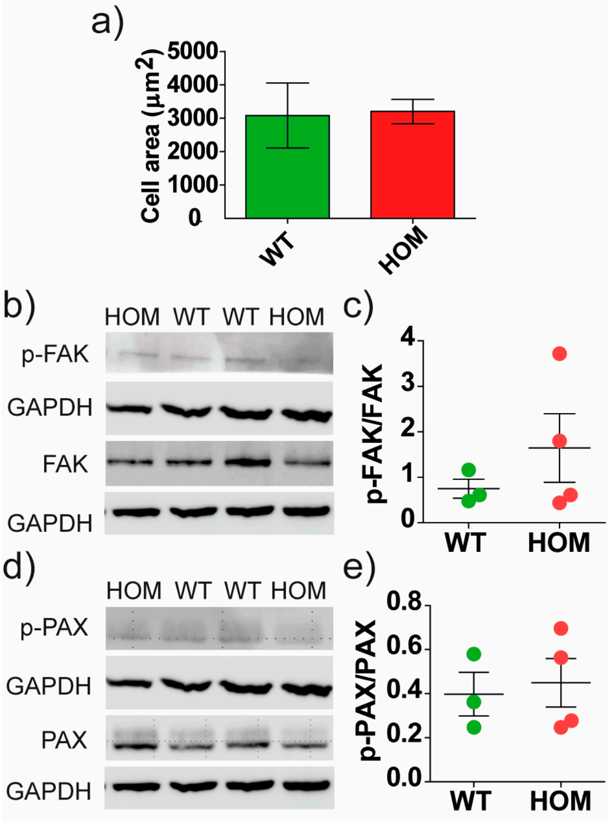

Figure S2. FAs intracellular pathway in WT and HOM fibroblasts. (a) Cell spreading area (µm^2) after 48h from seeding: cell size does not change, suggesting that there are no differences overall in the spreading and adhesion processes b-e) We performed western blot to analyse the activation and levels of FAK and Paxillin in fibroblast lysates. Representative western blot panels (b, d) and quantification of (c) phospho-FAK/FAK and (e) phospho-PAX/PAX levels in WT and HOM cells. Data = mean ± SEM, $n \geq 3$.

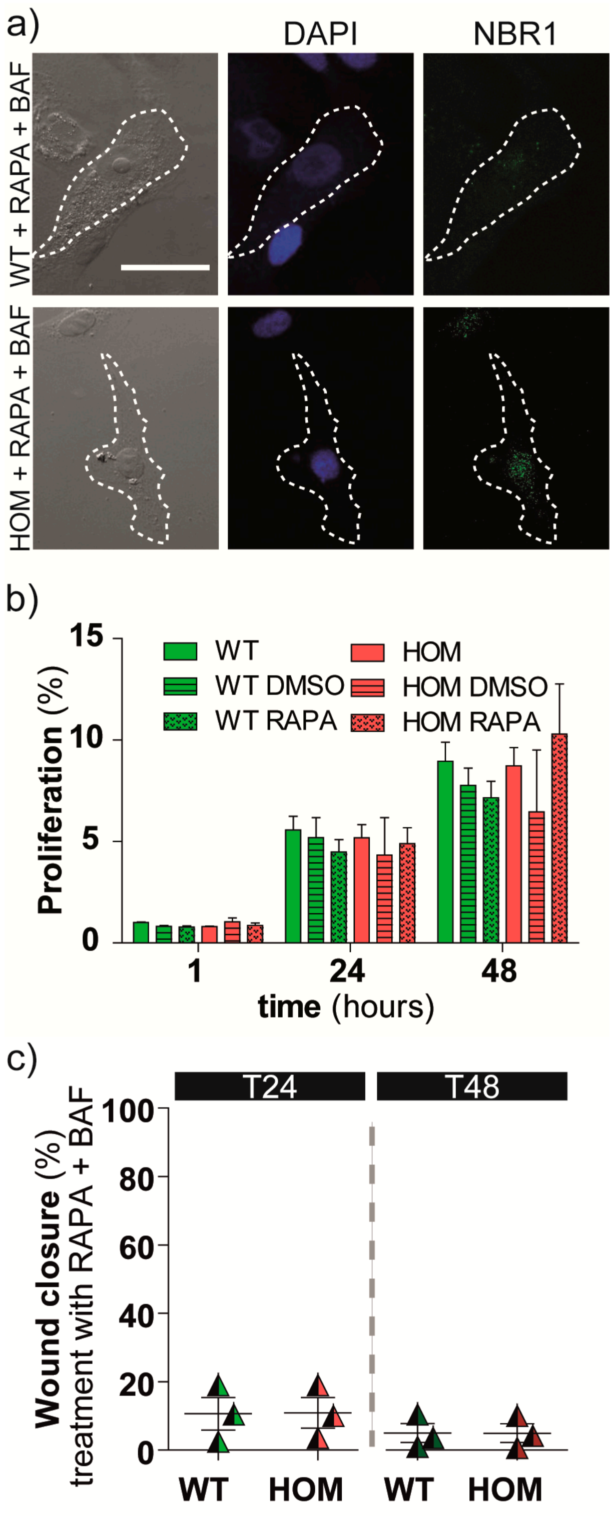

Figure S3. (a) Representative confocal images of WT and HOM twitcher fibroblasts treated with RAPA + BAF immunostained for (from left to right): bright field, nuclei (*blue*), NBR1 (*green*); scale bar: 50 µm. (b) Cell proliferation is reported as fold increase over the WT at t = 0 and measured at *t* = 0 – 24 – 48 hours. (c) Collective cell migration after BAF treatment: Wound closure (%) at *t* = 24 h (*left*, lighter colours) and *t* = 48 h (*right*, darker colours). Data = mean ± SEM, *n* = 3.

Samples	PSY Results (pg/µg protein)	n
**WT fibrobalsts**	< 9.1	n=4
	< 5.7	
	< 1.5	
	< 1.2	
**HOM fibroblasts**	< 3.5	n=4
	< 6.6	
	< 1.4	
	< 1.5	

Figure S4. PSY content in HOM and WT primary fibroblasts. Values are reported as the mean in pg/µg protein: the values that are lower than the detection limit of the system (< 18.2 pg/µg) are preceed by the < symbol. PSY levels were at very low and similar levels in both WT and HOM fibroblasts. PSY quantification was assessed on the cell lysates after a lipid extraction step, using Liquid Chromatography coupled with High-Resolution Mass Spectrometry (LC/HRMS), as previously done by us in [71]. In details, the extraction was carried out according to the method described by [72], with minor modifications. Briefly, each cell lysate (20-100 µL, containing at least 60 µg of proteins each) was diluted 4 times with a mixture 1:2 of Chloroform/Methanol containing the PSY-d5 as Internal Standard at the concentration of 200 ng/mL. Samples were mixed for 20 minutes on a thermomixer (Euroclone T-Shaker) at 1700 rpm and room temperature. Afterwards, proteins were sedimented by centrifugation at 13,000 rpm for 10 minutes at 4 °C. The supernatant was transferred into glass autosampler vials and immediately subjected to LC/HRMS analysis. A lipidomic liquid chromatography method was used to achieve a wide separation of PSY from other lipid molecular species. Quantitative data were obtained by injecting 20 µL of extract samples on an Agilent 6530 Q-TOF LC/MS (Agilent Technologies, Inc., Santa Clara, CA, USA), by Data Dependent Acquisition. Acetate adducts of natural PSY and PSY-d5 were monitored in negative ionization mode with accuracy within ± 2.5 ppm. The peak area was used for quantification. PSY concentration was expressed as pg/µg of total protein. Deuterated Psychosine Quantitative Mass Spec Standard (Galactosyl(β) Sphingosine-d5; PSY-d5) was purchased from Avanti Polar Lipids. Water, Acetonitrile, Isopropanol and Methanol LC/MS grade, Chloroform, Ammonium Acetate and Ammonium Fluoride were purchased from Sigma Aldrich.

Refs:

[71] Del Grosso, A., Parlanti, G., Angella, L., Giordano, N., Tonazzini, I., Ottalagana, E., Cecchini, M. Chronic lithium administration in a mouse model for Krabbe disease. JIMD reports 2021, 63, 50-65. doi: 10.1002/jmd2.12258

[72] Fuller M, Szer J, Stark S, Fletcher JM. Rapid, single-phase extraction of glucosylsphingosine from plasma: A universal screening and monitoring tool. Clin Chim Acta. 2015; 450:6-10. doi:10.1016/j.cca.2015.07.026
